# Supplementary material for: Association between Serum Phytosterols and Lipid Levels in a Population-Based Study
Source: Nutrients. 2024 Mar 8;16(6):775. doi: 10.3390/nu16060775 (PMC10975912; doi:10.3390/nu16060775)
Supplement: Supplementary file 1 [file nutrients-16-00775-s001.zip › nutrients-2886828-supplementary.pdf]

Supplemental material

Table S1: serum sterol concentrations according to gender, CoLaus|PsyCoLaus study, Lausanne, Switzerland.

	First survey			Second survey		
	**Women (N=538)**	**Men (N=372)**	**P-value**	**Women (N=434)**	**Men (N=287)**	**P-value**
Campesterol [mg/dl]	0.30 [0.19 - 0.44]	0.26 [0.17 - 0.37]	<0.001	0.26 [0.18 - 0.38]	0.26 [0.17 - 0.35]	0.281
Campestanol [µg/dl]	5.29 [3.39 - 8.02]	4.73 [3.10 - 7.15]	0.005	3.71 [3.07 - 4.61]	3.71 [3.09 - 4.49]	0.809
Stigmasterol [µg/dl]	5.55 [3.42 - 8.73]	5.02 [3.17 - 7.76]	0.020	7.33 [5.70 - 9.59]	6.76 [5.08 - 8.74]	0.006
Sitosterol [mg/dl]	0.24 [0.17 - 0.33]	0.21 [0.15 - 0.28]	<0.001	0.22 [0.17 - 0.31]	0.22 [0.16 - 0.29]	0.064
Sitostanol [µg/dl]	6.33 [4.64 - 9.29]	6.10 [4.42 - 8.14]	0.023	3.73 [3.24 - 4.47]	3.81 [3.32 - 4.44]	0.674
Brassicasterol [µg/dl]	17.4 [11.6 - 24.7]	17.0 [11.6 - 23.4]	0.253	18.2 [13.7 - 25.0]	19.0 [13.1 - 24.8]	0.954
Total cholesterol GC [µg/dl]	217 [193 - 245]	196 [173 - 220]	<0.001	198 [175 - 218]	178 [148 - 203]	<0.001

Results are expressed as median [interquartile range]. Between-sex comparisons performed using Kruskal-Wallis test.

Table S2: correlation coefficients between serum phytosterols and lipid markers, participants devoid of lipid-lowering drug treatment, CoLaus|PsyCoLaus study, Lausanne, Switzerland.

	**Total cholesterol**		**Total cholesterol GC**		**LDL cholesterol**		**HDL cholesterol**		**Triglycerides**		**ApoA-IV**	**Lp(a)**
	**First**	**Second**	**First**	**Second**	**First**	**Second**	**First**	**Second**	**First**	**Second**	**First**	**First**
Campesterol [mg/dl]	**0.365**	**0.357**	**0.378**	**0.294**	**0.308**	**0.253**	**0.262**	**0.280**	**-0.091**	**-0.120**	**0.083**	0.070
Campestanol [µg/dl]	**0.358**	**0.344**	**0.382**	**0.290**	**0.327**	**0.292**	**0.114**	**0.144**	**0.076**	0.022	0.062	**0.083**
Stigmasterol [µg/dl]	**0.261**	**0.315**	**0.253**	**0.239**	**0.222**	**0.244**	**0.098**	**0.172**	0.042	-0.050	**0.091**	0.059
Sitosterol [mg/dl]	**0.350**	**0.345**	**0.370**	**0.275**	**0.288**	**0.249**	**0.254**	**0.283**	**-0.087**	**-0.144**	**0.105**	0.075
Sitostanol [µg/dl]	**0.261**	**0.286**	**0.286**	**0.224**	**0.224**	**0.220**	**0.096**	**0.163**	0.060	-0.005	**0.080**	0.046
Brassicasterol [µg/dl]	**0.293**	**0.327**	**0.307**	**0.272**	**0.259**	**0.250**	**0.133**	**0.203**	0.008	-0.050	0.061	0.036

Results are expressed as Spearman nonparametric correlation coefficient. Data for the first (2009-2012) and second (2014-2017) follow-ups. Significant ($p<0.05$) results are indicated in bold.

Table S3: correlation coefficients between serum phytosterols and lipid markers, stratified by sex, CoLaus|PsyCoLaus study, Lausanne, Switzerland.

	Total cholesterol		Total cholesterol GC		LDL cholesterol		HDL cholesterol		Triglycerides		ApoA-IV	Lp(a)
	First	Second	First	Second	First	Second	First	Second	First	Second	First	First
**Male**												
Campesterol [mg/dl]	**0.340**	**0.202**	**0.327**	**0.176**	**0.270**	**0.257**	**0.273**	**0.141**	-0.015	-0.095	**0.117**	0.038
Campestanol [µg/dl]	**0.334**	**0.254**	**0.323**	**0.205**	**0.149**	**0.132**	**0.293**	**0.212**	0.076	0.014	0.027	0.048
Stigmasterol [µg/dl]	**0.205**	**0.202**	**0.203**	**0.176**	0.093	**0.140**	**0.172**	**0.189**	0.042	-0.083	0.089	-0.006
Sitosterol [mg/dl]	**0.309**	**0.250**	**0.300**	**0.230**	**0.256**	**0.232**	**0.259**	**0.213**	-0.066	-0.112	**0.127**	0.019
Sitostanol [µg/dl]	**0.214**	**0.134**	**0.201**	0.074	**0.145**	0.091	**0.177**	0.091	0.023	0.016	0.091	0.002
Brassicasterol [µg/dl]	**0.273**	**0.233**	**0.253**	**0.219**	**0.204**	**0.210**	**0.204**	**0.176**	0.040	-0.006	0.057	0.000
**Female**												
Campesterol [mg/dl]	**0.260**	**0.270**	**0.307**	**0.245**	**0.188**	**0.328**	**0.204**	**0.150**	-0.074	**-0.153**	0.073	0.025
Campestanol [µg/dl]	**0.320**	**0.275**	**0.386**	**0.268**	0.077	**0.173**	**0.281**	**0.217**	0.084	-0.028	0.036	0.051
Stigmasterol [µg/dl]	**0.223**	**0.236**	**0.247**	**0.187**	**0.094**	**0.217**	**0.163**	**0.156**	0.055	**-0.114**	0.082	0.045
Sitosterol [mg/dl]	**0.243**	**0.240**	**0.289**	**0.233**	**0.201**	**0.331**	**0.171**	**0.128**	-0.060	**-0.177**	0.073	0.042
Sitostanol [µg/dl]	**0.192**	**0.211**	**0.259**	**0.213**	0.039	**0.209**	**0.148**	**0.135**	**0.106**	-0.042	0.048	0.024
Brassicasterol [µg/dl]	**0.212**	**0.241**	**0.255**	**0.226**	0.084	**0.230**	**0.176**	**0.148**	0.019	-0.078	0.080	0.029

Results are expressed as Spearman nonparametric correlation coefficient. Data for the first (2009-2012) and second (2014-2017) follow-ups. Significant ($p<0.05$) results are indicated in bold.

Table S4: multivariable regression analysis between lipid and lipoprotein levels (dependent variable) and serum phytosterol levels, participants not treated for hyperlipidemia, CoLaus|PsyCoLaus study, Lausanne, Switzerland.

	**Total cholesterol**		**Total cholesterol GC**		**LDL cholesterol**		**HDL cholesterol**		**Triglycerides**	
	**First**	**Second**	**First**	**Second**	**First**	**Second**	**First**	**Second**	**First**	**Second**
Campesterol [mg/dl]	**1.645**	**1.348**	**62.4**	**71**	**1.606**	**2.237**	**0.231**	**0.551**	0.068	-0.006
	**(1.265; 2.025)**	**(1.002; 1.695)**	**(48.3 ; 76.6)**	**(50.5 ; 91.5)**	**(1.105; 2.106)**	**(1.708; 2.766)**	**(0.070; 0.392)**	**(0.298; 0.804)**	(-0.105; 0.241)	(-0.269; 0.257)
Campestanol [µg/dl]	**0.077**	**0.068**	**3.207**	**6.952**	**0.184**	**0.229**	-0.002	0.019	**0.016**	**0.038**
	**(0.058; 0.095)**	**(0.051; 0.084)**	**(2.529 ; 3.885)**	**(4.893 ; 9.012)**	**(0.134; 0.233)**	**(0.176; 0.282)**	(-0.010; 0.006)	(-0.007; 0.044)	**(0.008; 0.025)**	**(0.012; 0.064)**
Stigmasterol [µg/dl]	**0.060**	**0.051**	**2.085**	**1.921**	**0.051**	**0.062**	0.001	0.004	**0.012**	0.009
	**(0.041; 0.078)**	**(0.034; 0.067)**	**(1.39 ; 2.780)**	**(1.085 ; 2.757)**	**(0.031; 0.071)**	**(0.040; 0.084)**	(-0.007; 0.009)	(-0.006; 0.014)	**(0.004; 0.020)**	(-0.001; 0.019)
Sitosterol [mg/dl]	**2.465**	**2.009**	**94.6**	**84.2**	**2.138**	**2.694**	**0.296**	**0.494**	0.189	-0.020
	**(1.885; 3.044)**	**(1.481; 2.537)**	**(73.1 ; 116.1)**	**(57.7 ; 110.6)**	**(1.502; 2.773)**	**(2.010; 3.378)**	**(0.050; 0.541)**	**(0.166; 0.821)**	(-0.074; 0.452)	(-0.357; 0.318)
Sitostanol [µg/dl]	**0.049**	**0.043**	**2.25**	**3.209**	**0.093**	**0.123**	-0.003	0.018	**0.015**	0.017
	**(0.030; 0.068)**	**(0.026; 0.059)**	**(1.564 ; 2.935)**	**(1.428 ; 4.991)**	**(0.051; 0.136)**	**(0.077; 0.170)**	(-0.011; 0.005)	(-0.004; 0.040)	**(0.007; 0.023)**	(-0.005; 0.039)
Brassicasterol [µg/dl]	**0.024**	**0.019**	**0.882**	**0.945**	**0.020**	**0.029**	0.002	**0.007**	0.003	0.002
	**(0.017; 0.030)**	**(0.013; 0.025)**	**(0.632 ; 1.132)**	**(0.662 ; 1.228)**	**(0.013; 0.028)**	**(0.022; 0.037)**	(-0.001; 0.005)	**(0.003; 0.010)**	(0.000; 0.006)	(-0.001; 0.006)

Results are expressed as slope and (95% confidence interval). Data for the first (2009-2012) and second (2014-2017) follow-ups. Statistical analysis conducted by linear regression adjusting for age (continuous), sex (male, female), BMI (continuous) and diabetes (yes, no). Significant ($p<0.05$) associations are indicated in bold.

Table S5: multivariable regression analysis between lipid and lipoprotein levels (dependent variable) and serum phytosterol levels, stratified by gender, CoLaus|PsyCoLaus study, Lausanne, Switzerland.

	**Total cholesterol**		**Total cholesterol GC**		**LDL cholesterol**		**HDL cholesterol**		**Triglycerides**	
	**First**	**Second**	**First**	**Second**	**First**	**Second**	**First**	**Second**	**First**	**Second**
**Male**										
Campesterol [mg/dl]	**2.018**	**1.777**	**67.5**	**57.2**	**1.430**	**1.260**	**0.588**	**0.538**	-0.078	-0.147
	**(1.479 ; 2.557)**	**(1.071 ; 2.483)**	**(47.1 ; 87.9)**	**(29.2 ; 85.2)**	**(0.961 ; 1.900)**	**(0.620 ; 1.900)**	**(0.379 ; 0.796)**	**(0.224 ; 0.852)**	(-0.347 ; 0.191)	(-0.517 ; 0.223)
Campestanol [µg/dl]	**0.104**	**0.189**	**3.951**	**6.842**	**0.086**	**0.188**	0.004	0.033	**0.020**	0.017
	**(0.075 ; 0.133)**	**(0.136 ; 0.242)**	**(2.866 ; 5.036)**	**(3.501 ; 10.18)**	**(0.062 ; 0.111)**	**(0.113 ; 0.263)**	(-0.008 ; 0.016)	(-0.005 ; 0.071)	**(0.006 ; 0.034)**	(-0.027 ; 0.062)
Stigmasterol [µg/dl]	**0.05**	**0.043**	**1.786**	**1.414**	**0.039**	**0.043**	0.001	0.002	**0.015**	-0.003
	**(0.021 ; 0.079)**	**(0.012 ; 0.075)**	**(0.703 ; 2.869)**	**(0.177 ; 2.651)**	**(0.014 ; 0.064)**	**(0.015 ; 0.070)**	(-0.01 ; 0.012)	(-0.012 ; 0.016)	**(0.001 ; 0.029)**	(-0.019 ; 0.013)
Sitosterol [mg/dl]	**2.996**	**2.160**	**107.2**	**66.6**	**2.212**	**1.76**	**0.785**	**0.513**	-0.138	-0.322
	**(2.118 ; 3.874)**	**(1.241 ; 3.079)**	**(74.3 ; 140.1)**	**(30.2 ; 103.1)**	**(1.454 ; 2.970)**	**(0.944 ; 2.576)**	**(0.446 ; 1.125)**	**(0.102 ; 0.924)**	(-0.571 ; 0.295)	(-0.801 ; 0.156)
Sitostanol [µg/dl]	**0.070**	0.046	**2.544**	0.286	**0.058**	0.031	0.004	0.007	0.012	0.010
	**(0.039 ; 0.100)**	(-0.014 ; 0.106)	**(1.403 ; 3.684)**	(-2.078 ; 2.651)	**(0.032 ; 0.084)**	(-0.022 ; 0.084)	(-0.008 ; 0.016)	(-0.019 ; 0.034)	(-0.002 ; 0.027)	(-0.020 ; 0.041)
Brassicasterol [µg/dl]	**0.033**	**0.026**	**1.044**	**0.909**	**0.023**	**0.018**	**0.008**	**0.006**	0.002	0.002
	**(0.023 ; 0.043)**	**(0.017 ; 0.036)**	**(0.655 ; 1.434)**	**(0.527 ; 1.291)**	**(0.014 ; 0.032)**	**(0.009 ; 0.027)**	**(0.004 ; 0.012)**	**(0.002 ; 0.011)**	(-0.004 ; 0.007)	(-0.004 ; 0.007)
**Female**										
Campesterol [mg/dl]	**1.377**	**1.968**	**56.2**	**67.7**	**1.182**	**1.365**	0.097	**0.644**	0.131	-0.183
	**(0.976 ; 1.779)**	**(1.460 ; 2.476)**	**(41.2 ; 71.2)**	**(48.0 ; 87.5)**	**(0.805 ; 1.558)**	**(0.879 ; 1.851)**	(-0.080 ; 0.275)	**(0.39 ; 0.898)**	(-0.043 ; 0.305)	(-0.418 ; 0.051)
Campestanol [µg/dl]	**0.062**	**0.207**	**2.803**	**6.453**	**0.059**	**0.157**	-0.005	0.02	**0.015**	0.017
	**(0.045 ; 0.08)**	**(0.162 ; 0.252)**	**(2.153 ; 3.453)**	**(4.397 ; 8.509)**	**(0.042 ; 0.075)**	**(0.107 ; 0.206)**	(-0.013 ; 0.002)	(-0.007 ; 0.047)	**(0.007 ; 0.022)**	(-0.007 ; 0.041)
Stigmasterol [µg/dl]	**0.064**	**0.072**	**2.458**	**2.217**	**0.053**	**0.056**	0.004	**0.014**	**0.011**	0.002
	**(0.045 ; 0.083)**	**(0.05 ; 0.094)**	**(1.733 ; 3.182)**	**(1.364 ; 3.069)**	**(0.035 ; 0.071)**	**(0.035 ; 0.076)**	(-0.004 ; 0.013)	**(0.003 ; 0.024)**	**(0.002 ; 0.019)**	(-0.008 ; 0.012)
Sitosterol [mg/dl]	**2.028**	**2.304**	**81.2**	**86.6**	**1.677**	**1.731**	0.207	**0.647**	0.219	-0.216
	**(1.420 ; 2.635)**	**(1.623 ; 2.986)**	**(58.4 ; 103.9)**	**(60.3 ; 112.8)**	**(1.107 ; 2.248)**	**(1.087 ; 2.374)**	(-0.061 ; 0.475)	**(0.307 ; 0.986)**	(-0.044 ; 0.482)	(-0.525 ; 0.094)
Sitostanol [µg/dl]	**0.041**	**0.087**	**2.106**	**2.886**	**0.038**	**0.071**	-0.006	0.013	**0.014**	0.003
	**(0.023 ; 0.059)**	**(0.045 ; 0.129)**	**(1.438 ; 2.774)**	**(1.262 ; 4.511)**	**(0.022 ; 0.055)**	**(0.032 ; 0.110)**	(-0.013 ; 0.002)	(-0.008 ; 0.034)	**(0.007 ; 0.022)**	(-0.015 ; 0.022)
Brassicasterol [µg/dl]	**0.018**	**0.024**	**0.760**	**0.947**	**0.015**	**0.016**	0.000	**0.008**	**0.004**	-0.002
	**(0.011 ; 0.025)**	**(0.017 ; 0.032)**	**(0.500 ; 1.020)**	**(0.663 ; 1.230)**	**(0.009 ; 0.022)**	**(0.009 ; 0.023)**	(-0.003 ; 0.003)	**(0.004 ; 0.012)**	**(0.001 ; 0.007)**	(-0.005 ; 0.002)

Results are expressed as slope and (95% confidence interval). Data for the first (2009-2012) and second (2014-2017) follow-ups. Statistical analysis conducted by linear regression adjusting for age (continuous), BMI (continuous) and diabetes (yes, no). Significant ($p<0.05$) associations are indicated in bold.

Table S6: multivariable regression analysis between apolipoprotein A-IV, Lp(a) (dependent variable) and serum phytosterol levels, participants not treated for hyperlipidemia, CoLaus|PsyCoLaus study, Lausanne, Switzerland.

	**Apolipoprotein A-IV**	**Lp(a), log-transformed**
Campesterol [mg/dl]	0.705 (-1.137 ; 2.547)	0.192 (-0.290 ; 0.673)
Campestanol [µg/dl]	0.034 (-0.055 ; 0.123)	0.020 (-0.003 ; 0.043)
Stigmasterol [µg/dl]	-0.007 (-0.096 ; 0.082)	0.007 (-0.017 ; 0.030)
Sitosterol [mg/dl]	0.898 (-1.905 ; 3.701)	0.308 (-0.425 ; 1.041)
Sitostanol [µg/dl]	0.056 (-0.031 ; 0.143)	0.013 (-0.010 ; 0.036)
Brassicasterol [µg/dl]	0 (-0.033 ; 0.032)	0 (-0.008 ; 0.009)

Results are expressed as slope and (95% confidence interval). Data for the first follow-up (2009-2012). Statistical analysis conducted by linear regression adjusting for age (continuous), sex (male, female), BMI (continuous) and diabetes (yes, no). No significant ($p<0.05$) association was found.

Table S7: multivariable regression analysis between apolipoprotein A-IV, Lp(a) (dependent variable) and serum phytosterol levels, stratified by sex, CoLaus|PsyCoLaus study, Lausanne, Switzerland.

	**Apolipoprotein A-IV**	**Lp(a), log-transformed**
**Male**		
Campesterol [mg/dl]	**3.708 (0.631 ; 6.786)**	0.143 (-0.585 ; 0.871)
Campestanol [µg/dl]	-0.048 (-0.214 ; 0.119)	0.016 (-0.024 ; 0.055)
Stigmasterol [µg/dl]	0.062 (-0.096 ; 0.221)	0 (-0.037 ; 0.037)
Sitosterol [mg/dl]	**5.516 (0.556 ; 10.48)**	-0.073 (-1.245 ; 1.099)
Sitostanol [µg/dl]	0.068 (-0.100 ; 0.236)	-0.002 (-0.042 ; 0.037)
Brassicasterol [µg/dl]	0.039 (-0.019 ; 0.097)	-0.001 (-0.015 ; 0.013)
**Female**		
Campesterol [mg/dl]	1.564 (-0.408 ; 3.536)	0.010 (-0.475 ; 0.496)
Campestanol [µg/dl]	0.048 (-0.039 ; 0.134)	0.004 (-0.017 ; 0.026)
Stigmasterol [µg/dl]	0.025 (-0.070 ; 0.119)	-0.003 (-0.026 ; 0.021)
Sitosterol [mg/dl]	1.242 (-1.738 ; 4.222)	0.145 (-0.588 ; 0.877)
Sitostanol [µg/dl]	0.051 (-0.035 ; 0.137)	0.002 (-0.019 ; 0.023)
Brassicasterol [µg/dl]	0.023 (-0.011 ; 0.056)	0 (-0.008 ; 0.009)

Results are expressed as slope and (95% confidence interval). Data for the first follow-up (2009-2012). Statistical analysis conducted by linear regression adjusting for age (continuous), BMI (continuous) and diabetes (yes, no). Significant (p<0.05) associations are indicated in bold.
